# Molecular insights into pathogenesis and targeted therapy of peripheral T cell lymphoma

**DOI:** 10.1186/s40164-020-00188-w

**Published:** 2020-11-13

**Authors:** Caiqin Xie, Xian Li, Hui Zeng, Wenbin Qian

**Affiliations:** 1 Department of Hematology, The Second Affiliated Hospital, College of Medicine, Zhejiang University, 88# Jiefang Road, Hangzhou, 310009 Zhejiang People’s Republic of China; 2 Department of Hematology, First Affiliated Hospital of Jiaxing University, 1882# Zhonghuan South Road, Jiaxing, 314000 People’s Republic of China; 3 National Clinical Research Center for Hematologic Diseases, The First Affiliated Hospital of Soochow University, Suzhou, 215006 People’s Republic of China

**Keywords:** Peripheral T-cell lymphoma, Molecular genetic characteristics, Gene mutation, Targeted therapy

## Abstract

Peripheral T-cell lymphomas (PTCLs) are biologically and clinically heterogeneous diseases almost all of which are associated with poor outcomes. Recent advances in gene expression profiling that helps in diagnosis and prognostication of different subtypes and next-generation sequencing have given new insights into the pathogenesis and molecular pathway of PTCL. Here, we focus on a broader description of mutational insights into the common subtypes of PTCL including PTCL not other specified type, angioimmunoblastic T-cell lymphoma, anaplastic large cell lymphoma, and extra-nodal NK/T cell lymphoma, nasal type, and also present an overview of new targeted therapies currently in various stages of clinical trials.

Among malignant lymphomas, the peripheral T-cell lymphoma (PTCL) constitutes around 10–15% of all Non-Hodgkin’s lymphoma (NHL) in western countries, but, in Asian the proportion will increase to 20–30%. Currently, the World Health Organization (WHO) classification recognizes at least 29 subtypes of PTCL. Within this heterogeneous disease group, the most common subtype is PTCL not other specified type (PTCL-NOS), followed by angioimmunoblastic T-cell lymphoma (AITL), anaplastic large cell lymphoma (ALCL), and extra-nodal NK/T cell lymphoma, nasal type (ENKTL) [1]. In general, T-cell lymphomas have worse outcomes as compared to their B-cell counterparts. Most patients did not respond well to anthracycline-based CHOP (cyclophosphamide, doxorubicin, vincristine, and prednisone) chemotherapy, with 5-year overall survival (OS) of less than 30%. Patients with refractory recurrent (R/R) PTCL had a worse prognosis [2]. There are challenges in revealing distinct classes of genetic changes that occur in different subtypes of PTCL because relatively low incidence and the complexity of subtype classification. However, in recent years, the widespread application of molecular biology techniques, especially gene expression profiling (GEP) and next-generation sequencing (NGS) technology, has greatly improved our understanding of the genetic changes and pathogenesis of multiple subtypes of PTCL, which were successful in characterizing genetic alterations for AITL, ALCL, adult T-cell leukemia/lymphoma (ATLL), and PTCL-NOS [2]. In this review, we provide an overview on novel insights into molecular signatures, especially gene mutations and oncogenic pathways in four common subtypes of PTCL: PTCL-NOS, AITL, ALCL, and ENKTL, and also present an overview of new targeted therapies currently in various stages of clinical trials, but we exclude detailed descriptions for chemotherapy and hematopoietic stem cell transplantation, since these has been extensively reviewed elsewhere [3].

## Molecular signatures and oncogenic pathways in the common subtypes of PTCL

PTCL pathogenesis is a complex process that involves dysregulation of TCR pathways, malignant transformation driven by viruses and chronic inflammation, chromosomal alterations including translocations, insertions, deletions, and gene mutations. In addition, extensive epigenetic dysregulation plays a crucial role in tumor chemoresistance and disease progression. What follows is a description of some of the recent composite data for the major subtypes of PTCL.

### Peripheral T-cell lymphoma, not other specified type

PTCL not other specified type (PTCL-NOS), a highly heterogeneous entity, accounts for approximately ~ 30% of all PTCLs [4]. Recently, it is well known that a proportion (about 20%) of PTCL-NOS, similar to AITL, is derived from T-follicular helper (T_FH_) cells. Other cases of PTCL-NOS are derived from the TH1 or TH2 cells, and have similar gene expression profiles to cytotoxic T-cells, respectively [5]. The molecular findings in PTCL-NOS are heterogeneous and show significant overlap with other PTCL subtypes. Using GEP, two biological subgroups of PTCL-NOS have been identified, which include PTCL-GATA3 that is characterized by high expression of GATA3 and known target genes (CCR4, IL18RA, CXCR7, and IK), and PTCL-TBX21 that shows significantly higher expression of TBX21 (T-bet) and EOMES and their downstream targets (CXCR3, IL2RB, CCL3, IFN-γ) [6, 7]. The transcription factors TBX21 and GATA3 promote differentiation of CD4^+^ T cells into TH1 and TH2 helper cells, respectively [8]. The PTCL-GATA3 subgroup, accounting for 33% of PTCL-NOS, has an enrichment of MYC and cytotoxic gene with poorer outcome while the PTCL-TBX21 subgroup that constitutes 49% of PTCL-NOS, has a tumor microenvironment gene signature and is associated with a favorable outcome. However, there is a small subtype of TBX21-expressing PTCL that expressed cytotoxic gene-signature showing poor clinical a poor outcome [7].

**Fig. 1:**
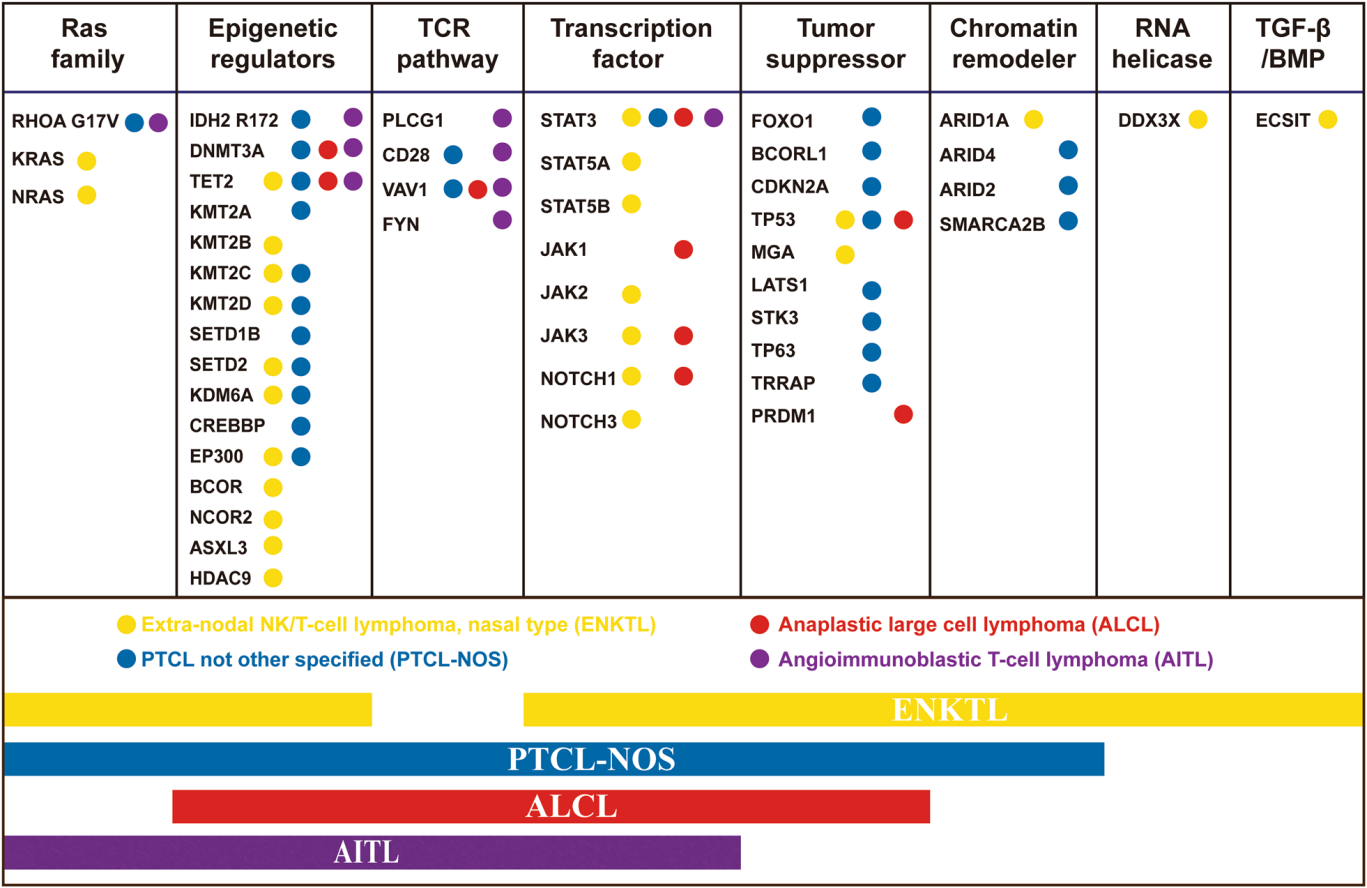
Landscape of somatic mutations in major subtypes of PTCL including PTCL not other specified type (PTCL-NOS), angioimmunoblastic T-cell lymphoma (AITL), anaplastic large cell lymphoma (ALCL), and extra-nodal NK/T cell lymphoma, nasal type (ENKTL), which shows distinct genetic features belonging to each functional pathway. Circles indicate the presence of mutation, yellow represents ENKTL while red, blue, and purple represent ALCL, PTCL-NOS, and AITL, respectively

Recent studies using NGS have unraveled the mutational landscape in PTCL-NOS leading to a marked improvement in the understanding of its pathogenesis and biology. Various recurrent genetic lesions have been identified in subsets of PTCL-NOS (Fig. 1). Class I genetic changes include mutations in epigenetic regulators. Among them, mutations of DNA methylation genes including TET2 (38–49%), DNMT3A (5–36%), and IDH2-R172 (0–8%) have been identified in patients with PTCL-NOS [9–13]. It was reported that mutant IDH2 and TET2 potentially cooperate with mutant RHOA to induce a potent T_FH_ phenotype in vivo [14]. In the PTCL patients with T_FH_ phenotype, recurrent TET2 mutations were associated with a worse prognosis [11]. Nevertheless, mutant TET2 was also observed at near-equal frequencies in PTCL-GATA3 and PTCL-TBX21. In addition, mutations in TET1, TET3, and DNMT3A were enriched in PTCL-TBX21 [2, 15], suggesting that the alterations in epigenetic regulators play an important role in pathogenesis of PTCL-TBX21 subtype. On other hand, mutations in regulators of histone methylation were observed in PTCL-NOS cases, which include MLL2 (4/28), KDM6A (3/28), MLL (2/28) [15], KMT2C and KMT2D [16]. Ji et al. [17] have demonstrated that mutations of histone methylation genes most frequently occurred in KMT2D (20%), followed by those in SETD2 (4.8%), KMT2A (2.4%) and KDM6A (0.8%). Meanwhile, loss-of-function mutations in histone acetylation genes were found in EP300 (8%) and in CREBBP (4%) that results in impaired p53 activation and promotion of oncogenic effects of Bcl-6 [18]. Chromatin remodeler genes ARID4, ARID2 and SMARCA2B were also affected [15, 17]. More importantly, patients with histone modifier gene mutations were associated with poor survival, whereas there was no predictive value of alterations of DNA methylation genes [15, 17]. The results from two independent studies showed that overlap mutations were seldom present among DNA methylation, histone methylation, histone acetylation, or chromatin remodeler genes [15, 17]. Together, alterations of histone modifying genes might represent a distinct biological characteristic. RHOA mutant (G17V), a loss-of-function mutation, has been identified in both AITL (53–71%) and PTCL-NOS (8–18%) [5, 19, 20]. As discussed above, the presence of RHOA (G17V) mutations in most PTCL-NOS cases correlated with a T_FH_ cell phenotype similar to the phenotype of AITL. However, the data from PTCL with T_FH_ phenotype suggest that the subtype may be a genetically distinct entity from AITL [2]. Interestingly, gene set enrichment analysis of PTCL-NOS and AITL cases with the mutant G17A compared to those with wild type RHOA revealed that there is a relationship between RHOA and Rac1 in which a high level of Rac activity leads to activation of multiple downstream signals, including the NFκB, p38 mitogen-activated protein kinase, and mitogen-activated protein kinase kinase/extracellular signal-regulated kinase pathways [21].

Class II genetic changes include mutations in tumor suppressor genes that include TP53, FOXO1, BCORL1, and ATM, albeit at lower frequencies [2, 13, 15]. Using whole exome sequencing alongside with RNA sequencing, Laginestra et al. [22] have demonstrated mutations of LATS1 (3/71), STK3 (4/71), ATM (11/71), TP53 (5/71), and TP63 (9/71). Among them, TP53 mutations were associated with PTCL-GATA3 while TP53 signaling genes (ATM and TRRAP) were mutated in both PTCL-GATA3 and PTCL-TBX21 [2]. Watatani et al. suggested there is a novel molecular subtype that is characterized by TP53/CDKN2A tumor suppressor inactivation in PTCL-NOS cases [13]. Similar to PTCL-GATA3, TP53/CDKN2A-altered PTCL-NOS has extensive chromosomal abnormalities, which were rarely seen in other groups, and is associated with an adverse outcome. In addition, the recurrent mutations in FAT1 tumor suppressor gene were found in 39% cases of PTCL-NOS, which is spanned over the two main GATA3/TBX21 subtypes. The patients with mutant FAT1 had a significantly reduced overall survival compared with cases without mutations, suggesting that mutations of FAT1 might be involved in the pathogenesis of PTCL-NOS [22].

Another genetic changes are recurrent somatic gene mutations, involving TCR signalling genes such as FYN, VAV1, and CD28. The mutant FYN, a gene encodes a tyrosine kinase that plays an important role in T-cell activation, was found in 3% of PTCL-NOS samples [13, 19, 23]. It was demonstrated a driver oncogenic role for VAV1 signaling in the pathogenesis of PTCL [24]. RHOA (G17V) binding to VAV1 increases TCR signaling [25]. Both Src-family kinases LCK and FYN are also involved in TCR signal transduction and T-cell activation [26], which are required for CD28 engagement and subsequent VAV1 phosphorylation [27]. In addition to CTLA-4-CD28 fusion genes and CD28 gain/amplification [2, 28], a recent study shows that CD28 mutations were present in PTCL-TBX21 subtype (5.3%) and AITL (11.3%) [13].

### Angioimmunoblastic T-cell lymphoma

Angioimmunoblastic T-cell lymphoma (AITL), the second most common subtype of PTCL, is derived from the T_FH_ cell, and has a distinct gene expression profile that is different from other PTCLs [5, 7, 29]. This PTCL entity had the least abnormal genome [2], and is quite well-defined with unique clinical characteristics, pathological features and aberrant oncogenic pathways including the NF-κB pathway, IL-6 signaling, and the TGF-β pathway that have been identified to be enriched in AITL [30]. Histologically, there are abundant endothelial venules in the tumor sample of AITL, which are differentiated from PTCL-NOS with T_FH_ phenotype [31].

Recent genetic advances have led to the discovery of recurrent mutations in AITL. TET2, IDH2, and DNMT3A are mutated in 76–83%, 20–45% and 26–38% of AITL cases, respectively [2, 9, 32–34]. These mutations of epigenetic regulators indicate a strongly dysregulated epigenome in AITL. TET2 mutations promote loss-of-function of the TET2 enzyme that is involved in DNA demethylation, resulting in DNA hypermethylation. In AITL, the lymphoma associated TET2 and DNMT3A mutations most likely occur at an early stage of hematopoiesis because these mutations are also present in non-tumoral hematopoietic cells of patients, thereby raising the possibility that they may be early events in oncogenesis [35]. The mutant TET2 contributes to AITL oncogenesis by not only increasing self-renewal capacity of hematopoietic stem/progenitor cells but up-regulating BCL-6 that alters T_FH_ differentiation [14]. DNMT3A and TET2 mutations were sometimes co-occurred in AITL. Odejide et al. [33]. reported that one-thirds of AITL cases have DNMT3A mutations and all patients with these mutations also harbored TET2 mutations. Similar to DNMT3A, mutations in IDH2 (R172) also appear to occur frequently with TET2 mutations [2, 10, 33, 34]. Lemonnier et al. [36] demonstrated that mutant IDH2 expression was restricted to the malignant T-cell component, and that 2-hydroxyglutarate (2HG) was elevated in lymphoma tissue and serum of AITL patients. Importantly, IDH2 (R172) mutations are usually confined to AITL and are rare in PTCL-NOS with T_FH_ phenotype [37, 38]. These mutations are also associated with chromosome 5 (Chr5) gain that is distinctive in AITL [2].

Recently, a multistep lymphomagenesis model was proposed, in which the premalignant hematopoietic progenitor cells harboring early mutations in epigenetic regulators TET2, DNMT3A are predisposed to the development of blood cancer, and the second-hit mutations such as RHOA (G17V) and IDH2 (R172) in a subclone of T_FH_ cells eventually leads to AITL [39–41]. RHOA (G17V) mutation is observed in 50–70% of AITL, which acts as a dominant-negative signaling protein, increasing cell proliferation by activation of AKT [10, 19, 20, 34]. Expression of RHOA (G17V) in CD4^+^ T-cells results in differentiations towards the T_FH_ lineages, suggesting an important role for this mutation in pathogenesis of AITL with T_FH_ features [42]. In AITL, RHOA (G17V) mutations are frequently associated with mutations of TET2 and IDH2 [10, 11, 19, 43]. The data from mouse model demonstrated that RHOA (G17V) cooperates with TCR stimulation to promote T_FH_-cell expansion, and lymphoma development in the presence of TET2 loss-of function [42, 44], suggesting the possibility of a functional interaction between these genetic alterations leading to a predilection for T_FH_ phenotype.

The genetic studies suggest the activation of TCR signaling by gene mutations may play a role in progression of AITL [45]. Actually, it was known that half of AITL have mutations in the TCR signaling pathways, including CD28 (13%), FYN (4.2%), PLCG1 (7.5%), STAT3 (3.1%), and VAV1 (4.3%) [31, 43]. The majority of mutations in TCR genes were activating and acted as a driver of lymphomagenesis [5]. RHOA (G17V) mutations have been reported to activate TCR pathway through direct binding to VAV1, an essential mediator of TCR signaling, and increasing its phosphorylation [25]. Mutant FYN also contributes to phosphorylation of downstream molecules such as PLCγ and VAV1 [46, 47]. Importantly, aberrant activation of VAV1 by RHOA (G17V) and VAV1 mutations are efficiently inhibited by dasatinib, a tyrosine kinase inhibitor. In a phase I clinical trial, 5 patients with relapsed/refractory AITL were treated with dasatinib, and all the evaluable patients achieved partial responses (PR), suggesting that AITL is highly dependent on the activation of TCR pathway [48]. Poor prognosis is reported for CD28 mutations that are more frequently observed in AITL than in PTCL-NOS [13, 31, 49]. The occurrence of the CTLA-4-CD28 fusion gene and CD28 mutations are mutually exclusive in AITL [41]. These two genetic changes activate intracellular signaling downstream of CD28 via PI3K/Akt/mTOR and Ras/Raf/MAPK/ERK signaling pathways, and result in increased survival and proliferation of T-cells.

Mutations in DNA methylation regulators including TET2, DNMT3A, and IDH2, as early events play an important role in multistage development of AITL [40]. However, these mutations are similar between AITL and myeloid neoplasms such as acute myeloid leukemia (AML) [50]. TET2 and IDH2 mutations were found to be mutually exclusive in AML. Whereas, IDH2 codon 172 (R172) mutations have been observed in ATIL, which often cooccur with TET2 mutations [2, 10, 33, 34]. Importantly, TET2 and IDH2 were associated with mutant RHOA status in AITL [10, 11, 19, 43]. On the other hand, IDH1/2 mutations that include IDH1 (R132), IDH2 (R140) and IDH2 (R172) are observed in about 20% in AML [51].

### Anaplastic large cell lymphoma

Anaplastic large cell lymphoma (ALCL), characterized by sheets of CD30 (Ki-1)-positive anaplastic large cells, is another of the more common PTCL subtypes, accounting for about 12% of all cases [8, 49]. Four distinct entities of ALCL, including ALK^+^ ALCL, ALK^−^ ALCL (represent 15–25% of non-cutaneous PTCLs in total), primary cutaneous ALCL (PC-ALCL), and breast prosthesis implantation ALCL (BI-ALCL), share common pathological features: a large-cell anaplastic morphology, strong CD30 expression and frequent phospho-STAT3 activation. In this section, we briefly review the molecular features of systemic ALCLs (sALCLs).

The sALCLs are usually aggressive nodal diseases. Among them, the ALK^+^ ALCL have a defining translocation featured by fusion of ALK, most commonly with NPM1 (85%), which have a significantly better outcome (70% to 90% 5-year OS) than ALK^−^ ALCL (15% to 58%) [52, 53]. However, there are many other fusion partners including TPM3, ATIC, TFG, TPM4, MYH9, RNF213, TRAF1, CLTC, and MSN, have also been reported [54]. The function of ALK fusion proteins is activation of multiple downstream signaling pathways, including PI3K/AKT, Ras/Erk, and Jak/STAT [54]. Recently, a new fusion of ALK with TRAF1 was found in patients with ALCL, which had an aggressive clinical course because the fusion leads to the constitutive activation of NFκB and acquisition of multiple genetic defects such as mutant TP53, the losses of PT53 and PRDM1/Blimp1 [55].

Recurrent rearrangements of non-tyrosine kinase genes also have been occurred in ALK^−^ ALCL, which include rearrangement of DUSPP22 and TP63. The patients with DUSP22 rearrangement (30%) showed good outcome similar to that of ALK^+^ ALCL while the patients with TP63 rearrangements (8%) presented a very poor prognosis with a 5-year OS rate of only 17% [56, 57]. Other structural alterations include ROS1, TYK2, VAV1, ERBB4, TP53, PRDM1 [57]. The chimeric proteins involved tyrosine kinases such as ROS1 and TYK2 can induce the activation of JAK/STAT pathway [58]. Losses of TP53 and PRDM1 were present in 52% ALK^−^ ALCL and may be associated with a more aggressive clinical course [31]. Compared to PTCL-NOS, ALK^−^ ALCL showed an enrichment of Myc and IRF4 target gene signature [7]. The expression of ERBB4, a member of the tyrosine kinase receptor superfamily, was found in approximately 25% ALK^−^ ALCL, but not in PTCL-NOS nor in ALK^+^ ALCL. Moreover, ERBB4 expression is mutually exclusive with other structural alterations such as TP63, DUSPP22 and ROS rearrangements or TYK translocations. ALCL with ERBB4 positive usually displayed Hodgkin-like features, thereby suggesting that there is a subset of ERBB4 expressing ALK^−^ ALCL [59, 60].

The genetics underlying ALCL at the level of somatic mutations remains unknown. Recently, sequencing studies have demonstrated the presence of recurrent mutations in systemic ALK^−^ ALCL, which include PRDM1/BLIMP1, TP53, STAT3, JAK1, and BANK1 [58]. Crescenzo et al. [58] demonstrated activating mutations of JAK1 and/or STAT3 genes in about 20% of ALK^−^ ALCL and showed that 38% of systemic ALK^−^ ALCL have double lesions. More importantly, JAK/STAT3 pathway inhibition significantly impaired cell growth in vitro and in vivo, suggesting that it was driver genetic alternations in ALK^−^ ALCL. Andersson et al. [61] discovered STAT3 mutations in 13% of AITL, 13% of ALK^+^ ALCL, 38% of ALK^−^ ALCL and 17% of PTCL-NOS. JAK1/3 mutations also observed in 15% of ALK^−^ ALCL, but not in other subtypes of PTCL. More recently, whole-exome sequencing studies confirmed that Notch pathways were enriched in mutations [62]. Among them, variant T349 of Notch1, which encodes one of the numerous EGF-like domains and confers growth advantage to T-cells, was detected in 12% of ALK^+^ and ALK^−^ ALCL. The γ-secretase inhibitors inhibited Notch1 thereby leading to cell death and co-treatment with ALK inhibitor Crizotinib induced synergistic antilymphoma activity [62].

### Extranodal NK/T cell lymphoma, nasal type

Extranodal NK/T cell lymphoma, nasal type (ENKTL), an aggressive lymphoma derived from NK cells or cytotoxic T cells with a strong association with Epstein Barr Virus (EBV), is prevalent in Asia including China, Central and South America [63, 64]. It was well known that multiple factors such as EBV infection, immunogenetic background, defective immune surveillance, and environmental factors have been implicated in the pathogenesis of ENKTL [64–67]. Results from comparative genomic hybridization (CGH) and loss of heterozygosity (LOH) analyses showed that the most common cytogenetic abnormalities are loss and gain of chromosomal copy number. Among them, deletions on chromosome 6q21 that resulted in downregulated expression of tumour suppressor genes located in the region (PRDM1, ATG5, AIM1, FOXO3, and HACE1) were frequently in ENKTL [68, 69]. Several recent studies using GEP have suggested that multiple oncogenic signaling pathway including NFκB, MAPK, and JAK–STAT pathway play crucial roles in the pathogenesis of ENKTL [65, 70, 71].

The NGS technologies have provided a catalog of recurrently mutated genes in ENKTL. Several studies revealed somatic mutations in genes regulating the epigenetic landscape, which included ARID1A, ASXL3, CREBBP, KMT2D (MLL2), KDM6A, EP300, BCOR and TET2 in NKTCL [72–75]. In these studies, KMT2D and BCOR were two of the most frequently mutated genes [75]. BCOR, an epigenetic regulator interacts with some histone-deacetylase-family genes while KMT2D encodes a histone methyltransferase, playing an important role in regulation of gene transcription. Gao and colleagues [74] reported that mutants KMT2D and TET2 were associated with the loss of protein expression and were also significantly associated with poor prognosis in NKTCL patients.

The data from GEP has revealed the upregulation of JAK/STAT pathway genes in ENKTL compared to normal NK cells [71]. In addition, JAK3, STAT3, and STAT5B mutations leading to the constitutive activation of the JAK/STAT pathway have been reported in ENKTL [75–79]. Xiong et al. [80] also demonstrated presence of JAK2, JAK3, STAT3, STAT5A, and STAT5B mutations/amplifications although their mutational frequency varies between different studies and ranges from 0 to 35% [67]. Other factors such as inactivation of PTPRK by gene deletion and aberrant promoter hypermethylation also results in constitutive activation of JAK/STAT pathway [81], suggesting the diverse molecular mechanisms underlying the activation of JAK/STAT pathway. In ENKTL, oncogenic activation of STAT3, NFκB, and overexpression of EBV latent membrane protein 1 (LMP1) induce the upregulation of programmed death ligand 1 (PD-L1), which plays a crucial role in immune evasion and correlates with poor prognosis [79, 82].

Another frequently mutated gene is DDX3X (12–20%), an RNA helicase gene [72, 83]. Inactivating mutations lead to cell cycle progression and activation of other pro-proliferative pathways such as NFκB pathway [72]. Mutations in tumor suppressor p53 and MGA have also been found at varying frequency [72, 75]. The NGS analyses show that mutations of P53 are present in approximately 11.8–16% of ENKTL cases [75, 83]. It was demonstrated that TP53 mutations have been associated advanced stage disease and may represent a secondary rather than an early oncogenic event. Moreover, ENKTL patients with mutations in DDX3X and TP53 had a much worse prognosis than patients without mutations in the two genes [72]. Lymphoma associated hemophagocytic syndrome (HPS), accounting for 40–50% of secondary HPS, is frequently observed in ENKTL [84]. The patients with ENKTL-associated HPS usually have the shortest median survival time (15–116 days) [84, 85]. Recently, Wen et al. demonstrated an ECSIT mutation (ECSIT-V140A) in 19% of ENTKL patients who had a higher incidence of HPS because of activation of NFκB pathway, release of pro-inflammatory cytokines, and promotion of macrophage activation [86].

More recently, integrated analysis of the genomic and transcriptomic features has been used to define three molecular subtypes of NK/T cell lymphoma, termed TSIM, MB, and HEA subtype [80]. TSIM NK/T cell lymphoma seem to arise from NK cells, whereas MB and HEA subtypes may arise from T cells. The characteristic lesions in TSIM subtype were mutations in JAK/STAT pathway (58.9%) and P53 (21%), as well as amp9p24.1/JAK2 locus, amp17q21.2/STAT3/5B/5A locus, amp9p24.1/PD-L1/2 locus (21.8%), and del6q21. The MB subtype mainly involved MGA mutation and 1p22.1/BRDT LOH) while the HEA subtype often has HDAC9, EP300, and ARID1A mutation. Importantly, this study shows that patients with these NK/T cell lymphoma subtypes have significantly different survival rates following treatment with pegaspargase-based regimens. Immunohistochemistry revealed that the incidence of positive expression for PD-L1 in tumor tissues were highest in the TSIM subtype, MYC in the MB subtype, and DAXX in the HEA subtype, indicating that molecular subtypes are sensitive to different targeted therapeutic strategies.

## New perspective in the targeted molecular therapy in PTCL

Currently, the treatment of systemic PTCL, the exception of ENKTL, is based on CHOP or CHOP-like chemotherapy; however, the relapse and refractory (R/R) disease is still frequently observed in patients, especially in high-risk patients [87]. l-Asparaginase-based chemotherapy becomes a standard of care for advanced, R/R ENKTL, but nearly 50% of patients with newly diagnosed ENKTL continue to experience disease progression [87, 88]. So far, no optimal therapeutic approach has been developed to treat PTCL patients and identification of molecular targets will improve the situation [87]. Here we provide a comprehensive overview of advances in targeted therapies in PTCL. Table 1 lists the recent clinical trials that have been conducted to improve the disease outcome.

**Table 1:** Current clinical trials used CHOP as backbone

Title	NCT Number	Conditions	Status	Interventions
Lenalidomide in combination with CHOP in patients with untreated PTCL	NCT04423926	PTCL, NOS AITL ALK^−^ ALCL Phase III–IV ALK^+^ ALCL EATL	Recruiting Phase 1/Phase 2	Drug: Lenalidomide Drug: Cyclophosphamide Drug: Doxorubicin Drug: Vincristine Drug: Prednisolone
Treatment of newly diagnosed peripheral T-cell lymphoma	NCT03631862	Newly diagnosed peripheral T-cell lymphoma	Recruiting Phase 4	Drug: Apatinib Drug: CHOP Regimen
Efficacy and safety of decitabine plus CHOP vs CHOP in patients with untreated peripheral T-cell lymphoma	NCT03553537	Peripheral T-cell lymphoma	Not yet recruiting Phase 3	Drug: Decitabine Drug: Cyclophosphamide Drug: Doxorubicin Drug: Vincristine Drug: Prednisone
CC486-CHOP in patients with previously untreated peripheral T-cell lymphoma	NCT03542266	Previously untreated peripheral T-cell lymphoma	Active, not recruiting Phase 2	Drug: CC-486 Administration Drug: CHOP Administration
Chidamide with CHOP regimen for de Novo PTCL patients (CHOP: cyclophosphamide, etoposide, vincristine and prednisone; PTCL: Peripheral T cell lymphoma)	NCT03268889	Peripheral T-cell lymphoma	Recruiting Not applicable	Drug: Chidamide
Clinical trial of chidamide combined with CHOP in peripheral T-cell lymphoma patients	NCT02809573	Peripheral T-cell lymphoma	Completed Phase 1	Drug: Chidamide Drug: cyclophosphamide Drug: adriacin Drug: vincristine Drug: prednisone
A Dose-Finding Study of Folotyn® (pralatrexate injection) Plus CHOP With peripheral T-cell lymphoma (PTCL)	NCT02594267	Peripheral T-cell lymphoma	Active, not recruiting Phase 1	Drug: Pralatrexate Injection
Compare efficacy of CHOP versus fractionated ICED in transplant-eligible patients with previously untreated PTCL	NCT02445404	Peripheral T-cell lymphoma	Recruiting Phase 2	Drug: CHOP Drug: fractionated ICED
Phase 1 dose finding study of belinostat for treatment of patients with peripheral T-cell lymphoma (PTCL)	NCT01839097	Peripheral T-cell lymphoma	Completed Phase 1	Drug: Belinostat Drug: CHOP
Efficacy and safety of romidepsin CHOP vs CHOP in patients with untreated peripheral T-cell lymphoma	NCT01796002	Peripheral T-cell lymphoma	Active, not recruiting Phase 3	Drug: Romidepsin + CHOP Drug: CHOP
ECHELON-2: a comparison of brentuximab vedotin and CHP with standard-of-care CHOP in the treatment of patients with CD30-positive mature T-cell lymphomas	NCT01777152	Anaplastic large-cell lymphoma Non-Hodgkin lymphoma T-cell lymphoma	Active, not recruiting Phase 3	Drug: brentuximab vedotin Drug: CHOP/CHP
CHOP vs GEM-P in 1st line treatment of T-cell lymphoma, multicentre phase II study	NCT01719835	Peripheral T-cell lymphoma NOS Anaplastic large cell lymphoma, ALK-negative Angioimmunoblastic T-cell lymphoma Hepatosplenic Gamma/Delta T-cell lymphoma Enteropathy-associated T-cell lymphoma	Active, not recruiting Phase 2	Drug: Cyclophosphamide Drug: Gemcitabine Drug: Doxorubicin Drug: Vincristine Drug: Prednisolone Drug: methylprednisolone Drug: Cisplatin
Intensive chemo-immunotherapy as first line treatment in adult patients with peripheral T-cell lymphoma	NCT01679860	Lymphoma, T-cell, peripheral	Completed Phase 2	Procedure: Clin A. CHOP-CAMPATH (Chemo-immunotherapy) + SCT Drug: Clin B (CHOP-CAMPATH) Chemo-immunotherapy
Treatment of peripheral T-cell lymphoma	NCT01664975	Peripheral T-cell lymphoma	Completed Phase 4	Drug: GDPT regimen Drug: CHOP regimen
Study of pralatrexate versus observation following CHOP-based chemotherapy in previously undiagnosed peripheral T-cell lymphoma patients	NCT01420679	Peripheral T-cell lymphoma	Terminated Phase 3	Drug: Pralatrexate injection
A phase 3 trial of E7777 in combination with CHOP compared with CHOP alone for the first-line treatment of peripheral T-cell lymphoma	NCT01355783	Peripheral T-cell lymphoma	Withdrawn Phase 3	Drug: E7777
A study of escalating doses of romidepsin in association with CHOP in the treatment of peripheral T-cell lymphomas	NCT01280526	Peripheral T-cell lymphoma	Completed Phase 1/Phase 2	Drug: Romidepsin and CHOP
RAD001 combined with CHOP in newly diagnosed peripheral T-cell lymphomas	NCT01198665	Peripheral T-cell lymphoma unspecified Anaplastic large cell lymphoma, ALK-negative Angioimmunoblastic T cell lymphoma Cutaneous T cell lymphoma	Completed Phase 1/Phase 2	Drug: RAD001 (Everolimus)
Safety study of darinaparsin in combination with cyclophosphamide, doxorubicin, vincristine, and prednisone (CHOP) to treat lymphoma	NCT01139359	Lymphoma	Withdrawn Phase 1	Drug: darinaparsin Drug: CHOP
Endostar combined with CHOP regimen as first line chemotherapy for peripheral T cell lymphoma	NCT00974324	T cell lymphoma	Unknown Phase 2	Drug: endostar and CHOP
The effectiveness of alemtuzumab given in combination with CHOP and ESHAP in patients newly diagnosed with peripheral T-cell lymphoma (PTCL)	NCT00930605	Peripheral T-cell lymphoma	Completed Phase 2	Drug: CHOP regimen alternate with ESHAP regimen Drug: Alemtuzumab
CD4 in combination with CHOP in treating non-cutaneous peripheral T cell lymphoma	NCT00893516	T-cell Lymphoma	Terminated Phase 2	Biological: CHOP + CD4 Drug: CHOP
SAHA + CHOP in untreated T-cell non-Hodgkin's lymphoma	NCT00787527	Lymphoma	Completed Phase 1/Phase 2	Drug: Zolinza (vorinostat) Drug: Cyclophosphamide Drug: Doxorubicin Drug: Vincristine Drug: Prednisone
Immunotherapy in peripheral T cell lymphoma—the role of alemtuzumab in addition to dose dense CHOP	NCT00725231	Peripheral T cell lymphoma, unspecified Angioimmunoblastic lymphadenopathy Extranodal NK/T-cell Lymphoma	Unknown Phase 3	Biological: alemtuzumab Drug: chemotherapy
Alemtuzumab and CHOP in T-cell lymphoma	NCT00646854	Lymphoma, T-cell, peripheral	Completed Phase 3	Drug: CHOP14 chemotherapy (cyclophosphamide, hydroxydaunorubicin, vincristin, prednison) plus G-CSF, combined with alemtuzumab Drug: CHOP14 chemotherapy (see specification under Arm B) plus G-CSF
Alemtuzumab and CHOP chemotherapy for aggressive histological peripheral T-cell lymphomas	NCT00453427	Peripheral T-cell lymphomas	Completed Phase 1/Phase 2	Drug: Alemtuzumab (Campath-1H)
The effectiveness of alemtuzumab combination with CHOP to treat patients newly diagnosed with PTCL	NCT00441025	Peripheral T-cell lymphoma	Terminated Phase 2	Drug: Alemtuzumab
Bortezomib and CHOP in patients with advanced stage aggressive T cell or natural killer (NK)/T cell lymphomas	NCT00374699	Peripheral T-cell lymphomas Non-hodgkin lymphoma	Completed Phase 1/Phase 2	Drug: Velcade
A pilot study to determine the safety of the combination of Ontak in combination with CHOP in peripheral T-cell lymphoma	NCT00337987	Peripheral T-cell lymphoma	Completed Phase 2	Drug: Ontak Drug: CHOP (cyclophosphamide (C), adriamycin (H), vincristine (O), and prednisone (P)) chemotherapy
A study of ONTAK and CHOP in newly diagnosed, peripheral T-cell lymphoma	NCT00211185	Lymphoma, T-cell, peripheral	Completed Phase 2	Drug: Denileukin diftitox Drug: Cyclophosphamide Drug: Doxorubicin Drug: Vincristine Drug: Prednisone Other: Pegfilgrastim

The detail show on https://clinicaltrials.gov/

### Targeting cell surface molecules

Surface antigens are the most accessible part of lymphoma cells, and histologic characterization of specific PTCL subtypes has provided several cell surface markers that can be targeted for disease-specific treatment strategies. For example, CD30 shows variable expressions in different PTCL subtypes. There is nearly 100% expression of CD30 in ALCL regardless of ALK expression, and CD52 can be detected by flow cytometry in 35% to 100% of PTCL-NOS cases [89].

Brentuximab vedotin (BV) is an antibody that link to an anti-tubulin agent monomethyl auristatin E (MMAE), which targets CD30-expressing cells, disrupting the microtubule network, causing cell cycle arrest and apoptosis. Previous clinical studies showed that BV had significant activity in CD30-expressing ALCL, with durable remission. Moreover, in a global double-blind, randomized phase III study, BV plus CHP (CHOP without vincristine) demonstrated higher complete remission (CR) rate and superior overall survival relative to CHOP as frontline treatment for patients with PTCL. However, non-ALCL cases such as PTCL-NOS enrolled in this study are less than 25% [8]. Kim et al. [90] reported that the disease control rate was 48.5% in the patients with R/R high CD30-expressing NHL including PTCL-NOS (24.2%), ENKTL (21.2%) and AITL (3%). Recently, Wagner et al. [91] suggested that a combination of bendamustine and BV may be an effective salvage therapy in patients with PTCL. Together, the data from clinical trial suggest that BV has opened a new era in the management of PTCL.

Alemtuzumab, a humanized mAb targeting CD52, can induce complement-mediated lysis as well as apoptosis in malignant lymphoid cells. PTCL subtypes such as PTCL-NOS and AITL were shown to have high frequency of CD52 expression (> 90%) by flow cytometry and immunohistochemistry (IHC) analysis, while CD52 expression was low in ALCL and ENKTL. Alemtuzumab has been used in the setting of untreated aggressive T and NK cell lymphomas as in combination with chemotherapy, showing a high rate of CR, but with an unacceptable safety profile [92, 93]. The DSHNHL2006-1B/ACT-2 trial reported that Alemtuzumab added to CHOP increased response rates in elderly PTCL patients, but did not improve survival due to treatment-related toxicity [94].

Daratumumab is a CD38 mAb approved for treating R/R and untreated multiple myeloma. Because CD38 is universally expressed within ENKTL, Daratumumab has been recently investigated to assess its safety and efficacy within ENKTL (NCT02927925), which shows a promising overall response rate (ORR) of 35.7% in 16 patients [8, 95].

Mogamulizumab (KW-0761), a defucosylated, humanized monoclonal antibody targeting CCR4, has direct cytotoxic effect on CCR4-positive lymphoma cells via antibody-dependent cellular cytotoxicity, as well as immunomodulatory potential by depletion of regulatory T cells to enhance anti-tumor immunity. The CCR4 is expressed in approximately 30–65% of PTCL tumor cells, including high expression (~ 65%) in ALK^−^ ALCL, variable expression (30–40%) in PTCL-NOS, AITL, and transformed mycosis fungoides (MF), while rarely expressed in ENKTL or ALK^+^ ALCL [96]. Mogamulizumab is approved by the FDA for the treatment of R/R Sezary syndrome and MF [96]. In a multicenter phase 2 trial, 37 relapsed PTCL and cutaneous T-cell lymphoma patients were treated with mogamulizumab. The ORR for PTCL-NOS and AITL were 19% and 50%, respectively [97].

### Immune checkpoint inhibitor for PTCL

The PD-L1 are frequently expressed in AITL and PTCL with T_FH_ phenotype. In ALK^+^ tumor cells, activation of the STAT3 driven by NPM-ALK fusion protein results in the increased expression of PD-L1 [98]. The expression of PD-L1 has also been reported in ENKTL with a frequency of 67%, which is presumably secondary to EBV infection [79, 82]. Several clinical trials of PD-1 blockade treatment in PTCLs have been conducted. A phase II study comprising patients with R/R PTCL-NOS (41%) and T cell lymphoma with T_FH_ phenotype (18%) demonstrated an ORR of 33%, and four patients achieved CR [99]. Furthermore, in a phase II study with pembrolizumab, 7 patients with ENKTL were treated, 4/7 patients achieved an objective response, including two CR in this study [100].

### CAR-T therapy in PTCL

The chimeric antigen receptor T (CAR-T) cells are produced by transducing a genetically engineered CAR fusion protein that targets cell surface on tumor cells. Many promising avenues are being explored toward translating CAR-T therapy for the treatment of PTCLs [101]. Recently, CAR-T cells directed against CD30 have been developed and are being explored in clinical trial for treating R/R CD30^+^ PTCL patients [102]. Ramos et al. [103] treated 9 patients with R/R CD30^+^ lymphoma using CD30-CAR-T cells, in which 1/2 ALCL patients achieved CR that persisted 9 months. CD7, a transmembrane glycoprotein, is expressed in majority of lymphoblastic T-cell leukemia and in a subset of PTCL [104]. However, frequent loss of CD7 is observed in tumor cells of PTCL [105]. Recently, two clinical trials using CD7-specific CAR-T cells were launched to assess the safety and efficacy in the patients with CD7^+^ NK/T cell lymphoma (NCT04004637) and T-NHL (NCT04033302), respectively. The development of CAR-T therapy for T-cell malignancies has been limited by a lack of target antigens that discriminate tumor cells from normal T cells, which will run the risk of severe or unmanageable immunosuppression [101]. Interestingly, Maciocia et al. [106] developed a new strategy for the CAR-T therapy to treat T-cell malignancies and demonstrated that anti-T cell receptor-chain constant domain 1 (TRBC1) CAR-T recognized and killed normal and malignant TRBC1^+^, but not TRBC2^+^, T cells in mouse model of leukemia. The phase I/II study that evaluate the safety and efficacy of TRBC-specific CAR-T is ongoing (NCT03590574).

### Targeting the epigenome therapy

In PTCLs, epigenetic dysregulation has been shown to have a major role in the pathogenesis of PTCL-NOS, AITL, and ENKTL subtypes (Fig. 1), resulting in the use of molecular inhibitors designed to target epigenetic mechanisms in clinical practice.

Belinostat is a pan-class I and II HDAC inhibitor that induces apoptosis and cell cycle arrest in abnormal, transformed cells through complex interactions with cell cycle mechanisms. Belinostat was tested in a phase 2 study in patients with R/R PTCL including PTCL-NOS (64.2%), AITL (18.3%), and ALK-ALCL (10.8%), and showed an ORR of 25% [107]. Romidepsin, a structurally unique, potent, bicyclic class I selective HDAC inhibitor, has demonstrated durable clinical responses in patients with R/R PTCL, leading to its approval by the US Food and Drug Administration (FDA) in 2011 for the treatment of PTCL in patients who have received at least one prior therapy [108]. Chidamide is a novel benzamide class of HDAC inhibitor, selectively inhibiting activity of HDAC class I, II, III, and X, which was the first approved orphan drug for PTCL in China [108]. In a recent phase II study, R/R PTCL patients who received chidamide alone had an efficient disease control. The ORR was 28%, including 14% CR/unconfirmed CR (CRu). The patients with AITL showed higher ORR (50%) and CR/CRu rate (40%), as well as more durable response [109]. Consistent with this result, a large retrospective study shows that HDAC inhibitor may have superior activity in T_FH_-PTCL compared with non-T_FH_-PTCL [110].

The hypomethylating agents 5-azacytidine and decitabine show efficacy in myeloid neoplasms such as AML, and the response rate to these drugs appears to correlate with TET2*, *IDH1/2, and/or DNMT3A mutations. Lemonnier et al. [111] reported 12 AITL patients who received 5-azacytidine for concomitant myeloid neoplasm or used as compassionate treatment in R/R AITL. Nine patients (75%) exhibited a response, including 6 CR. Among them, 5 patients have shown a sustained response, as they are still in CR 23 months after treatment. Interestingly, TET2 mutations were detected in all patients while 4/12 (33%) patients had DNMT3A mutations and 5/12 (41%) patients had RHOA mutations.

### Other targeted therapy

It is now increasingly clear that there are different aberrant signal pathways for each of these different subtypes of PTCL, which highlight new targeting concepts.

Crizotinib is an inhibitor of multiple tyrosine kinases, including the ALK. Gambacorti Passerini et al. [112] reported the long-term follow-up of crizotinib administered to 11 ALK^+^ lymphoma patients that included 9 patients with R/R ALCL. The ORR was 10 of 11 (90.9%) and included 9 CR (81.8%) and 1 PR. In pediatric ALCL patients, crizotinib demonstrated robust and sustained clinical responses [113].

Duvelisib is an oral inhibitor of phosphatidylinositol 3-kinase (PI3K)-δ/γ isoforms currently in clinical development [114]. A phase I trial of Duvelisib in 16 patients with R/R PTCL produced an ORR of 50%, with 3 CR and 5 PR [114]. ENKTL and AITL frequently show oncogenic activation of JAK/STAT pathway that can be targeted by a variety of molecule inhibitors. Ruxolitinib, a JAK1/2 inhibitor, is now being investigated in phase II clinical trials for patients with relapsed ENKTL (NCT02974647) [67]. Dasatinib, a multi-kinase inhibitor, can inhibit the Src family tyrosine kinases including FYN and LCK. A phase I clinical trial of dasatinib in 5 patients with AITL were performed and showed a promising result with an achievement of PR in all evaluated patients [115].

### Combinatorial targeted therapy

Recently, combinatorial strategies have been tested to improve the efficacy of targeted therapy [108]. Based on the synergism between HDAC inhibitors and hypomethylating agents in preclinical models, O'Connor et al. [116] performed a phase 1 clinical study of 5-azacytidine and romidepsin among R/R lymphoma patients. This combination produced OR and CR rates of 73% and 55%, respectively in patients with T cell lymphoma. Ghione et al. [110] conducted a multicenter retrospective cohort study and analyzed the outcomes of patients with PTCL (AITL, T_FH_-PTCL, and PTCL-NOS) treated with HDAC inhibitor, either in combination (duvelisib, carfilzomib, or lenalidomide) or as single agents. When HDAC inhibitors were used as single agent, ORR and CR were 45.3% and 23.7%, respectively while ORR and CR in the combination group were 46.6% and 30%, respectively. Importantly, patients with T_FH_-PTCL had higher ORR when compared with PTCL-NOS (71% vs 29%), but had similar ORR when those with AITL (71% vs 56%).

## Summary

The applications of NGS technology in the last decade, have significantly improved our knowledge on the genetic and molecular of PTCL, which may eventually lead to identification of better treatment options and provide a deeper understanding of the disease pathogenesis and potential therapeutic targets in the future. Treatment remains a challenge for PTCL. However, new agents or novel rational drug combinations targeting specific pathways in patients with PTCL will hopefully lead not only to better control of the disease, but also to the additional improvements in outcome for patients with R/R disease.

## Data Availability

Not applicable.
